# Deep Learning Based Over-the-Air Training of Wireless Communication Systems without Feedback

**DOI:** 10.3390/s24102993

**Published:** 2024-05-08

**Authors:** Christopher P. Davey, Ismail Shakeel, Ravinesh C. Deo, Sancho Salcedo-Sanz

**Affiliations:** 1 School of Mathematics, Physics and Computing, University of Southern Queensland, Springfield, QLD 4300, Australia; 2 Spectrum Warfare Branch, Information Sciences Division, Defence Science and Technology Group (DSTG), Edinburgh, SA 5111, Australia; ismail.shakeel@defence.gov.au; 3 Department of Signal Processing and Communications, Universidad de Alcalá, 28805 Alcalá de Henares, Madrid, Spain; sancho.salcedo@uah.es

**Keywords:** deep learning, feedback-free training, trainable wireless communications systems, over-the-air training, neural networks

## Abstract

In trainable wireless communications systems, the use of deep learning for over-the-air training aims to address the discontinuity in backpropagation learning caused by the channel environment. The primary methods supporting this learning procedure either directly approximate the backpropagation gradients using techniques derived from reinforcement learning, or explicitly model the channel environment by training a generative channel model. In both cases, over-the-air training of transmitter and receiver requires a feedback channel to sound the channel environment and obtain measurements of the learning objective. The use of continuous feedback not only demands extra system resources but also makes the training process more susceptible to adversarial attacks. Conversely, opting for a feedback-free approach to train the models over the forward link, exclusively on the receiver side, could pose challenges to reliably end the training process without intermittent testing over the actual channel environment. In this article, we propose a novel method for the over-the-air training of wireless communication systems that does not require a feedback channel to train the transmitter and receiver. Random samples are transmitted through the channel environment to train a mixture density network to approximate the channel distribution on the receiver side of the network. The transmitter and receiver models are trained with the resulting channel model, and the transmitter can be deployed after training. We show that the block error rate measurements obtained with the simulated channel are suitable for monitoring as a stopping criterion during the training process. The resulting method is demonstrated to have equivalent performance to the end-to-end autoencoder training on small message sequences.

## 1. Introduction

**Figure 1:**
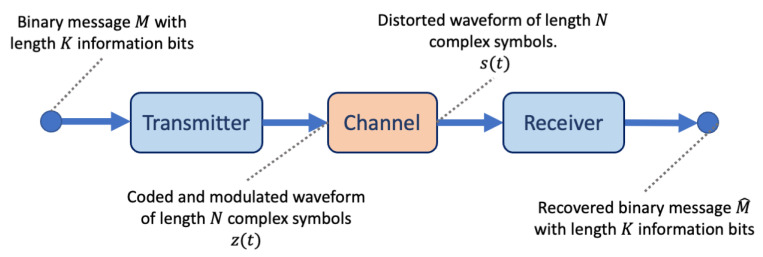
A simplified view of a wireless communications system. The transmitter takes in a *K* bit binary message *M* and codes and modulates the message producing transmitter symbols z(t). These symbols are transmitted over a channel that produces noise and outputs symbols s(t). The receiver is responsible for correcting the channel distortions and producing an estimate for the original message $\hat{M}$.

Messages in a wireless communication system are sent from a transmitter over the air, via a channel environment, to a receiver whose aim is to recover the original message. A simplified depiction of such a wireless communications system is shown in Figure 1. The channel environment is significant in this type of communications system, as it distorts the message with perturbations such as noise and fading effects. These channel effects, along with imperfections within the electronics of both the transmitter and the receiver, present a challenge to the recovery of the original message. To improve accuracy at the receiver, the transmitter can code message bits to enable error correction at the receiver. It is also responsible for modulating the message bits and converting the modulation to a radio frequency (RF) signal suitable for sending over the wireless channel. At the receiver, the distorted RF signal must be detected, demodulated, and decoded in order to recover the original sequence of bits. Each of these steps is conventionally defined as separate signal processing blocks that are optimised independently of one another [1].

End-to-end deep learning (DL) for wireless communications systems has been proposed as an alternative design approach to that of block-based traditional design [1]. The primary advantage of DL over block-based design is the potential to perform end-to-end optimisation over observations of a complex channel environment. Especially where the channel environment may be too complex to be expressed mathematically. However, in [1], the channel environment is assumed and described by a differentiable channel transfer function that does not necessarily capture the description of a true channel environment. Instead of assuming a channel function, it is preferable to jointly optimise the DL-based transmitter and receiver over examples produced by the true channel. However, in DL this poses a genuine challenge. The backpropagation algorithm, which modifies the parameters of the model, cannot occur between the transmitter and receiver. This is because the calculation of the gradient for the model parameters cannot be determined without a differentiable channel function.

To overcome this limitation, over-the-air learning (OAL) methods have either applied gradient approximation [2,3] or trained a separate generative channel model to enable end-to-end backpropagation [4,5]. The primary limitations of gradient approximation are the requirement to sample several perturbations through the channel at each training iteration and continuous feedback of the receiver error. Continuous feedback increases channel usage and exposes the training process to eavesdropping and data poisoning attack by adversary communications systems [6]. In the generative channel modelling approach, the generative adversarial network (GAN) has been widely adopted to approximate the wireless channel distribution [4,5,7]. However, GAN training requires two models to learn a channel approximation, a generator and a discriminator model, and proceeds in two stages. First, by training the discriminator to recognise true channel symbols versus those produced by the generator model, and second by training the generator to fool the discriminator. This adversarial training regime adds complexity to the overall training process for the transmitter and receiver.

In our prior work, we proposed a disjoint OAL algorithm that trains the transmitter with a local receiver by imitating the errors made at the remote receiver [8]. The local receiver relied on a feedback channel to supply the remote error information. However, as in gradient approximation, the feedback channel increases channel use and is vulnerable to eavesdropping and data poisoning attack during training.

Reliance on continuous feedback is a vulnerability for the overall security of OAL of DL-based wireless communications systems. To realise OAL on energy-constrained devices such as in the internet of things (IoT), it is important to avoid complex training procedures which require training multiple models. Both of these considerations motivate the work in this article with the following aims:

- To simplify the training procedure for OAL learning of transmitter and receiver, by proposing an alternative to gradient approximation and eliminating the requirement for the use of a feedback channel, as well as by developing a simple channel model that does not require adversarial training against a discriminator, while still learning an accurate approximation of the observed channel distribution.
- To reduce the vulnerability of OAL training to eavesdropping and adversarial attacks by removing the use of a feedback channel and preventing transmission of information-carrying symbols over the true channel environment that could be intercepted and altered during training.

Motivated by these challenges, in this article, we investigate a method for OAL in wireless communication systems that can be performed on the receiver side. The proposed approach does not require continuous feedback and requires training only a single model to approximate the distribution of the true channel. Additionally, we determine that intermittent evaluation of the transmitter and receiver using the resulting channel model is a suitable method for determining training stopping criteria and provides a measurement appropriate for monitoring of the learning process.

The key contributions of this article are:

- We propose an iterative OAL algorithm for the development of a transmitter, receiver, and channel model § that does not require continuous feedback between transmitter and receiver.
- We discuss the application of the mixture density network (MDN) for the approximation of the channel transfer function. We also show the demonstration of approximation for several simulated channels, including the additive white Gaussian noise (AWGN), Rician fading, Rayleigh fading, and power amplifier AWGN channels.
- We capture the simulated block error rate (BLER) for transmitter and receiver models measured over the generative channel model and demonstrate that this measurement correlates well with the BLER measured over the true channel environment, thereby showing that the simulated BLER is suitable for use as the training stopping criteria and for monitoring of the learning process.
- Finally, we demonstrate that the performance of the resulting transmitter and receiver models is equivalent to or better than the end-to-end model that is trained with an assumed channel model. This is shown for AWGN, Rician fading, Rayleigh fading, and non-linear power amplifier distortions over AWGN simulated channels, thereby matching the performance of more complex OAL methods that compare against the end-to-end model in the literature.

In this article, we present the background for end-to-end learning and related work in Section 2. In Section 3, we describe the system model and our proposed approach. We present and discuss results for the proposed approach compared with the end-to-end method in Section 4. In Section 5, we discuss limitations and simplifying assumptions for the proposed method and describe how these may be addressed in Section 6.2, which describes avenues for Future Work. Finally, we summarise our findings and conclude our paper in Section 6.1.

## 2. Background and Related Work

The most commonly cited motivation for the use of DL in training a wireless communication system is for its potential as a data-driven method to jointly optimise both the receiver and transmitter with respect to the distortions produced from the channel [1,4,5,7,9,10,11,12,13,14]. This motivation has spurred much investigation into the practical considerations required to realise the goal of automated design. Notably, the end-to-end design was first presented in [1], which demonstrated the application of the autoencoder (AE) model to the end-to-end joint optimisation using backpropagation for the transmitter and receiver over an assumed channel. The AE structure is divided into an encoder or transmitter component, a differentiable channel transfer function, and a decoder or receiver component. It is demonstrated to learn an encoding that can produce a BLER similar to the conventional Hamming(7,4) code over the AWGN channel [1]. The backpropagation training of wireless communications systems suffers a significant flaw, however, and that is the requirement for end-to-end differentiation must also assume a differentiable channel transfer function. This limitation prevents the design method from being applied in physical channel environments.

The simplest way to address this limitation is to take a two-step approach: first, training the end-to-end system offline, and second, performing tuning of the receiver model in the true channel environment. This procedure is demonstrated in [9], with a more realistic channel function that includes upsampling, timing, phase, and frequency offsets. Incorporating these additional distortions in the channel function required additional design considerations in the receiver model, which included a data preprocessing step to slice windows of the incoming signal, a phase estimation, and general feature extraction layers whose outputs were concatenated to feed into the receiver classifier [9]. The transmitter and receiver architectures were trained end-to-end, and the receiver was tuned post-deployment in both simulated AWGN and physical channels. The performance of the AE did not quite match the conventional differential quadrature phase-shift keying (QPSK) modulation but did demonstrate the first practical application of end-to-end training to OAL. Joint optimisation of both the transmitter and receiver models remained elusive, however, since only the receiver benefited from tuning in the deployed channel environment.

Gradient approximation methods were developed to enable optimisation for both the transmitter and receiver in OAL without prior knowledge of the channel. Two notable approaches were developed, the first being derived from simultaneous perturbation stochastic approximation (SPSA) [2] and the second based on Reinforcement learning (RL) policy gradient methods [15]. Both methods require that the transmitter outputs are perturbed multiple times to sample the loss from the receiver at several small displacements around the transmitter outputs [2,15]. The SPSA method requires more sampling than the latter method and does not scale well to longer messages or more complex transmitter models [3]. Both approaches did, however, demonstrate the feasibility of the method and achieved performance equivalent to the joint end-to-end approach in AWGN and Rayleigh fading channels. Subsequent work has advanced the use of the RL-based approach with application to concatenated coding and demonstrating good performance on longer message sequences, which addresses the short message limitation for symbol-wise classification in end-to-end learning [10]. However, reliance on the feedback channel increases channel use and the vulnerability to data poisoning during training, and multiple forward passes through the transmitter in each single training epoch can be avoided with an appropriate proxy channel model.

A DL channel model can be applied to learn the physical channel environment directly from observations without assuming a model for the true channel. Once trained, the channel model acts as a proxy to support backpropagation in the end-to-end training for transmitter and receiver models. GAN training methods have been adopted for their ability to approximate a distribution given noisy inputs. A variational AE generator was applied in [5] to receive transmitter outputs and approximate the channel distribution for several channels. The variational AE generator maps the transmitter symbols to the parameters for an internal normal distribution and uses samples from the inner distribution to map into the channel distribution. This method enables the model to approximate the stochastic quality of the channel [5]. The method is shown to approximate several channels, including AWGN, a non-Gaussian Chi-squared channel effect, and a non-linear channel over AWGN, which includes a hardware amplifier [5]. While this article demonstrated the potential application for modelling channels using the GAN, it did not consider how to apply the resulting channel model in the end-to-end training regime.

Instead of sampling with a variational AE, the context information produced by transmitting pilot symbols was applied to help the generator approximate the channel function in [4]. The resulting conditional GAN is trained on simulated AWGN and Rayleigh fading channels, and then used as a proxy for the true channel to train the transmitter and receiver [4]. The resulting performance was very close to the Hamming(7,4) code on the AWGN channel and was similar to coherent detection in the Rayleigh fading channel [4]. Refs. [4,5] train the AE with the adversarial learning algorithm where a separate discriminator aims to differentiate between true and generated samples and the generator aims to fool the discriminator into misclassifying generated samples [4]. However, one problem in adversarial training is that the generator model can suffer from mode collapse, where it confines generated results to a smaller area of the broader distribution to consistently fool the discriminator and subsequently fails to perform generalisation in modelling the extent of the target distribution [16].

The Wasserstein generative adversarial network (WGAN), which modifies the adversarial loss function, is proposed to improve training stability and address the issue of mode collapse for GAN training [17]. A WGAN model is trained on the receiver side without the need for continuous feedback in [7]. The target data set is first constructed by using a pre-trained transmitter to send a batch of transmissions through the channel. Once the batch has been collected, the WGAN can be trained with adversarial learning, and the resulting generator can be used to train a transmitter and receiver end-to-end. Instead of applying symbol-wise decoding, the approach used bit-wise decoding in a manner similar to [10]. The authors demonstrated one of the first instances where the GAN approach was applied in a physical channel to train the transmitter and receiver. However, when experimenting with the more dynamic time delay channel, the WGAN did not converge due to mode collapse, indicating that generative methods are challenged when learning more complex channels [7].

A conditional GAN that is trained on both transmitter symbols and received pilot symbols is proposed in order to generate more complex time-varying channel distributions in [11]. The method extends the work in [4] to longer codes using convolutional neural network (CNN) layers and proposes an iterative training algorithm for transmitter, GAN, and receiver. By including the pilot symbols as well as the transmitter symbols, the generator model is able to more closely match the channel effects observed during training [11]. Evaluation of the resulting system in simulated AWGN, Rayleigh fading, and frequency-selective fading channels demonstrates similar performance to that of an end-to-end AE trained with an assumed channel. However, the transmitter, channel, and receiver models are trained in an iterative manner [11], indicating a high channel usage during the training procedure similar to the RL method.

Rather than generating the channel distribution directly, the authors in [13] use a residual connection to learn the distribution of the differences between transmitter symbols and the received symbols output by the channel. The method residual aided generative adversarial network (RA-GAN) is trained on simulated channel data via an iterative training scheme and evaluated against a GAN-based model [13]. Evaluation in the AWGN, Rayleigh fading, and a ray-tracing-based channels demonstrates performance close to the optimal end-to-end AE training scheme and is close to the performance for both WGAN- and RL-based methods [13]. The approach simplifies the structure of the GAN, as well as introduces an additional regularisation term. However, the approach shares the same disadvantage as the other GAN-based training methods.

Each of the GAN-based methods requires a separate discriminator neural network that is used to train the generator during the adversarial training procedure. Adversarial training is a two-step procedure where the discriminator is first trained to classify true channel observations versus the generated samples, and secondly, the discriminator is used to train the generator to produce samples closer to the true observations [11]. After training the channel model, the discriminator is discarded. However, if considering training OAL on embedded IoT devices, there will be limitations to the capabilities of hardware platforms, unlike host driven software defined radio (SDR). It is preferable to reduce the number of models, which each requires training iterations; therefore, a single-channel model that can accurately approximate the channel distribution is preferable.

Difficulty with training stability for the GAN model has been quoted as a motivation for the different variations that have been applied in the literature [4,7,13]. An alternate single-channel model, the diffusion-denoising probabilistic model (DDPM). is adopted in [14], primarily to address the issue of mode collapse in the GAN method and because it has shown excellent performance in the image generation domain. The DDPM learns the parameters for the variance of a forward noising process where Gaussian noise is repeatedly added starting from the original input, and a reverse process which learns to restore the original data from the noise [14]. However, the denoising procedure is slow, requiring multiple recursive steps, hence a variation of the approach denoising diffusion implicit model (DDIM) is proposed to trade-off between accuracy and time [14]. Two approaches of training are adopted for comparison: the pre-trained approach trains the channel generator model before using it in the end-to-end training procedure, and the iterative approach interleaves training of channel generator, transmitter, and receiver [14]. Evaluation of the trained transmitter and receiver models is carried out with a K=4, N=7 code in the simulated AWGN, Rayleigh fading, and non-linear amplifier AWGN channels [14]. Pre-training was demonstrated to have the closest performance to the original end-to-end training method, and 50 iterations for the DDIM method was shown to be a good trade-off between accuracy and speed in comparison to the DDPM approach [14]. While diffusion models have demonstrated excellent generative capabilities in the image domain, the number of iterations to perform denoising adds to the latency during training, which is a disadvantage for the application of this approach to OAL. The advantage of the GAN is that after training, the channel can be simulated with a single forward pass. However, the training complexity due to adversarial learning against a discriminator model is the primary limitation for GAN-based methods in OAL. Therefore, a generative model that does not require multiple passes to reconstruct the signal and that supports a simple training regime is desirable for applications that may operate on embedded devices over a physical channel environment.

MDNs combine conventional neural networks with a mixture density model to learn an underlying generative mapping between input and target data [18]. The MDN trains a neural network to approximate general distributions by learning the parameters for a Gaussian mixture model [18]. In this manner, it is trained using conventional supervised learning without the need for a discriminator or multiple applications of noise and is a much simpler modeling framework than the GAN or diffusion-denoising models. A standard network can be seen as learning the mean of the target mapping through the least-squares loss, and the MDN instead models the parameters for the distribution of multi-valued continuous target variables [18]. This advantage over standard neural networks makes the MDN suitable for use in optimization problems, which may include non-unique solutions for different parameters [19]. This has led to the application of MDN to parameter estimation for inverse problems [20,21,22] and to simulation of physical processes [23].

Parameter estimation in the wireless environment is especially challenging due to noise and fading as well as other distortions such as timing, frequency, and phase offsets. However, the MDN has been demonstrated to enable accurate estimation for localisation of wireless sensor network devices in an environment featuring both AWGN and fading effects in [20]. The MDN has also been demonstrated to provide accurate approximation for the distributions of latency measurements taken in a 5G wireless AWGN environment [21]. In a related domain, the MDN was applied to the estimation of direction of arrival for acoustic signals also within an AWGN environment, and was shown to capture an accurate model of the uncertainty due to the channel [22]. In the radar domain, the MDN is demonstrated as an effective data-driven method to approximate radar sensor measurements for distance, velocity, and orientation of a moving vehicle [23]. In this scenario, a transmitted chirp signal is distorted by channel perturbations and noise as well as fading and the Doppler effect [23].

In this article, we propose a method for OAL without feedback, thereby reducing the channel use and opportunity for data poisoning attacks. A MDN channel model is trained by observing transmitted random uniform noise over the true channel environment. The MDN can be trained in a supervised manner to approximate the true channel distribution without use of a discriminator for adversarial training and is able to learn without the need of multiple forward passes or repeated noise correction in each epoch.

## 3. Methodology

### 3.1. System Model

In our work, we assume a single input single output (SISO) wireless communications system. A *K* bit binary message *M* is coded with an *N* bit code and modulated at the transmitter to produce a set of complex transmitter symbols z∈C. Experiments are carried out with K=8 bits and N=8 symbols. The transmitter symbols are transferred over the wireless channel, which we simulate as a transfer function r(t)=h(z(t)). The channel adds noise and other perturbations such as fading. In this article, training is carried out at a fixed signal to noise ratio (SNR) of 6 dB, and evaluation is performed over the SNR range of 0 dB to 15 dB. The receiver is responsible for detecting the signal, correcting distortions, demodulation, and decoding to produce an estimate of the original message $\hat{M}$. In our system, we assume perfect synchronisation; therefore, we do not add additional effects such as time delay, phase, or carrier frequency offset. The set of channels that are applied in this article are described in Section 3.5.

The developed AE-based transmitters and receivers learn to produce an uncoded modulation. Therefore, we include the BLER for uncoded binary phase shift keying (BPSK) to provide a reference for the optimal performance of an uncoded modulation. The difference in the performance is due to the ability of the AE to learn to utilise the entire in-phase and quadrature (IQ) space in the learnt constellation as opposed to using only two symbols available to BPSK modulation.

### 3.2. Joint End-to-End Approach

**Figure 2:**
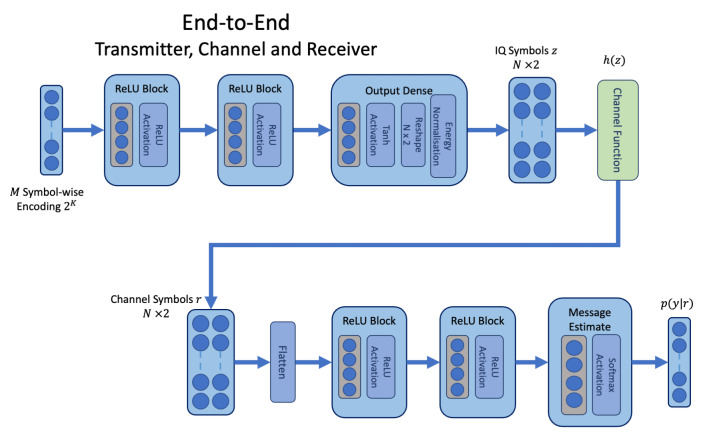
The end-to-end network architecture where an assumed channel transfer function is defined as a layer within the network architecture.

The joint end-to-end approach, based on the AE from [1], is depicted in Figure 2. This approach is trained end-to-end and incorporates a differentiable channel function h(y) as part of the model. The transmitter inputs consist of the one-hot encoded vector for the *K* bit message *M*. The one-hot encoding indicates the *i*th message as a one in $i\in 2^{K}$ index positions, where all other positions j≠i are set to 0. The output at the receiver is a vector of $2^{K}$ probabilities p(y|r) conditioned on channel symbols *r* where $\sum_{i=1}^{2^{K}}p\left(y_{i}|r\right)=1$. This is facilitated by the softmax activation $p\left(y_{i}|r\right)=exp\left(l_{i}\right)/\sum_{j}^{2^{K}}exp\left(l_{j}\right)$ where *l* is learnt by the receiver neural network. The index for the maximum probability is mapped to the corresponding index of the original message $M_{\mathrm{index}}=\arg\;\max\;p\left(y|r\right)$. Under this regime, the end-to-end model is trained against the cross-entropy (CE) loss shown in Equation (1). $p(y_{true})$ is represented as the one-hot encoding for the true messages and p(y|r) is softmax output produced by the receiver. In our work, we consider this joint model the baseline AE model, which has assumed knowledge of the channel environment, and we compare our proposed method to this model.

(1) $$L\left(p\left(y_{true}\right),p\left(y|r\right)\right)=-\sum_{i=1}^{2^{K}}p\left(y_{i}\right)\log p\left(y_{i}|r\right)$$

In the literature for OAL methods, the joint end-to-end AE based on [1] serves as the baseline comparative method. This is because, under simulation, the assumed channel function provides the joint end-to-end AE with complete information of the simulated environment and hence provides the optimal performance for the DL-based method. To demonstrate the effectiveness of the proposed method our aim is to demonstrate equivalent performance, since our proposed method does not have complete information about the channel, it must rely on training a proxy model of the true channel environment to learn an optimal constellation for that environment.

### 3.3. Proposed Approach

**Figure 3:**
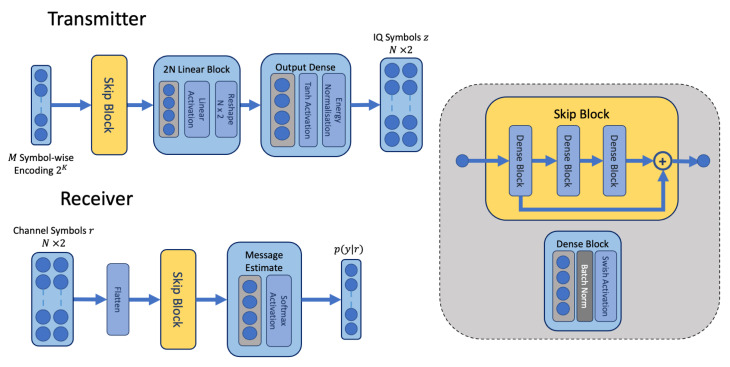
The architecture of the transmitter and receiver networks containing the dense residual skip block for feature extraction.

The transmitter and receiver blocks in our proposed approach differ from the original AE in [1]. Instead of dense blocks, we define a residual block with skip connections between the dense units, illustrated in Figure 3. The skip block in our architecture (shown in Figure 3) consists of three dense blocks consisting of linear units, batch normalisation [24] and a swish activation [25]. The first block scales incoming features so that they have a compatible dimension for addition to the output of the final block. Skip connections, also known as residual connections, mitigate vanishing gradients in deeper networks and are indicated to form an ensemble of models by combining multiple paths through the network [26]. Any number of skip blocks may be arranged in sequence in the network architecture. In our model, we typically used one skip block per transmitter and receiver network. Our choice of the swish activation is related to our choice of skip connections. The ReLU activation is known to suffer from a vanishing gradient due to its exclusion of negative values [27]. We chose the swish activation function to help mitigate the vanishing gradient and complement the use of the skip blocks to aid in promoting learning during backpropagation. Experimentally we have found the swish activation to outperform ReLU activations, as indicated in [25]. Since the swish activation is unbounded for positive values, batch normalisation is applied to reduce the impact of extremes in the activation values.

The *L* symbols, output by the transmitter, are scaled to emulate the energy constraint of the transmitter hardware such that ${\left||x|\right|}_{2}^{2}\leq 1$, shown in Equation (2). The tanh activation function is applied at the output of the transmitter to ensure that the learnt transmitter symbols remain within the range [−1,1]. Instead of an assumed channel function, backpropagation is enabled by the channel model, which is trained to approximate the true channel during the proposed training procedure (described in Section 3.4). The channel model connects transmitter and receiver models and acts as the proxy for the true channel to allow training to take place on the receiver side. This allows the training to occur without the need for feedback over the true channel, thereby reducing the opportunity for data poisoning during the training of the transmitter and receiver. Once trained, the transmitter weights can be transferred to the origin transmitter side to send messages across the true channel. Table 1 and Table 2 indicate the respective network dimensions for the transmitter and receiver.

(2) $$z\left(t\right)=\frac{x(t)}{\sqrt{\sum_{i=1}^{L}x{\left(i\right)}^{2}/L}}$$

**Figure 4:**
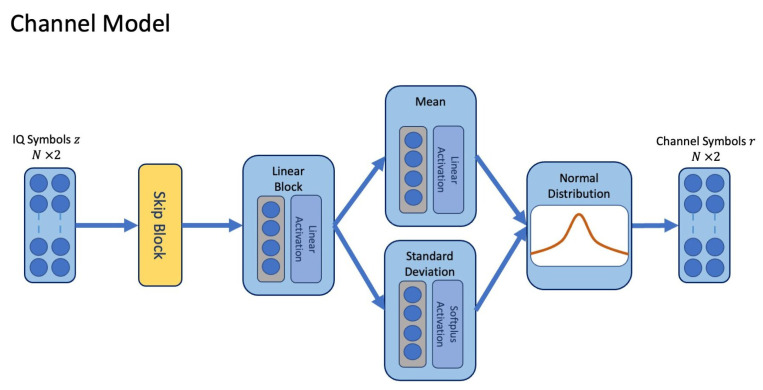
The channel model estimates parameters for the mean and standard deviation of a normal distribution for each transmitted symbol, and the estimate for channel effects is sampled from the resulting distribution.

To train the transmitter and receiver, we apply a channel MDN model. The channel MDN model is trained in a supervised manner against observations of noise transmitted through the true channel. Unlike the GAN, it does not require a separate discriminator model and does not require multiple denoising steps in comparison to the diffusion modelling approach. The resulting channel model is combined with the transmitter and receiver during an end-to-end learning phase, where it emulates the true channel environment. The MDN model estimates parameters for the mean and standard deviation $\theta =\left\{\mu_{j},\sigma_{j}\right\}$ and mixing coefficients $\phi_{j}$ for j=1…J Gaussian distributions for each individual symbol z(t) in *L* time-steps [18]. The resulting mixture of Gaussian distributions is combined to generate a probability density over the channel outputs p(r(t)|z(t)) (Equation (3)). In our implementation, each symbol may have a different mean and standard deviation, which are produced by the main path of the network consisting of a skip block and dense linear block illustrated in Figure 4.

Our model is a simplification of the MDN since we use only one Gaussian distribution and do not model coefficients [18]. However, we do learn separate mappings for each time-step from z(t) to θ. While it is possible to extend the modelling approach to include more than one set of Gaussian distributions, we found that estimating an individual mean and variance for each IQ symbol was sufficient for the set of channels used in the evaluation.

(3) $$p\left(r\left(t\right)|z\left(t\right)\right)=\sum_{j=1}^{J}\phi_{j}\left(z\left(t\right)\right)N\left(\mu_{j}\left(z\left(t\right)\right),\sigma_{j}^{2}\left(z\left(t\right)\right)\right)$$

The network is trained by minimising the negative log-likelihood (NLL) loss, shown in Equation (4), where $r{\left(t\right)}_{\mathrm{true}}$ are the true channel responses, z(t) are the transmitter symbols, and θ are the distribution parameters learnt by the MDN. A linear activation is applied to the estimate for the mean, and a softplus activation [28] is added with a small positive constant for the standard deviation. While this model is simpler than other generative approaches such as the GAN and diffusion models, it performs well in enabling the transmitter and receiver to learn modulation and coding that produces equivalent or better BLER when compared with the end-to-end learning approach. Table 3 lists the dimensions for each of the layers in the channel model.

(4) $$L\left(r{\left(t\right)}_{true},z\left(t\right),\theta \right)=-\ln\left\{p(r{\left(t\right)}_{true}|z\left(t\right),\theta )\right\}$$

### 3.4. Training Procedure

**Figure 5:**
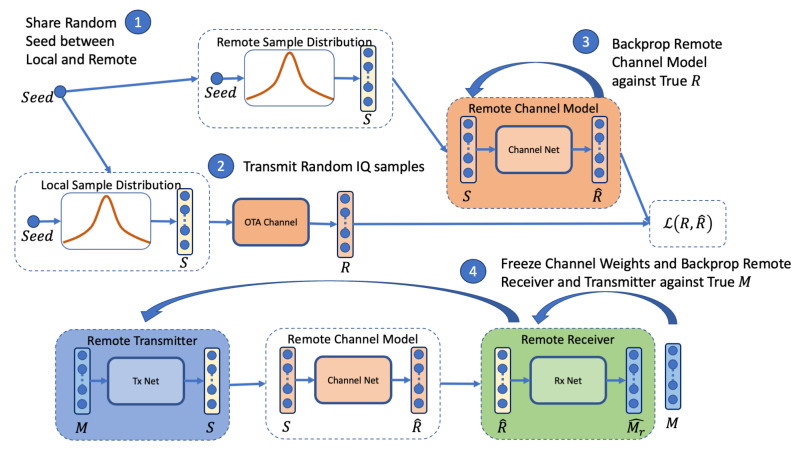
Schematic view of the proposed OAL procedure, without feedback. Stage 1 samples random values from the uniform distribution using a shared seed. Step 2 transmits the random values *S* over the true channel to receive channel symbols *R*. Step 3 trains the remote channel model on input *S* and back propagates against true channel symbols *R*. In Step 4, the channel model weights are frozen, and training via backpropagation for message *M* is performed using the remote channel model as a proxy for the true channel.

An overview of the training procedure is illustrated in Figure 5, where in stage 1, the initialisation of the training procedure requires that both an origin transmitter and remote receiver share the same random seed. This is used to draw continuous IQ samples of desired block length *K* from the uniform distribution S∼U([−1,1]). We emphasise that the random sequence *S* is not an information-carrying modulation. In stage 2, the random sequence is transmitted from the origin transmitter to the remote receiver, producing channel symbols *R*. A batch size of 128 blocks is collected prior to training the channel model. Backpropagation is applied in stage 3 to train the remote channel model against the true channel symbols *R* using the NLL loss. Stage 4 performs end-to-end training of the transmitter and receiver on the receiver side using the trained channel model, without the need for a feedback channel.

In stage 4, the weights of the channel model are frozen so that they are not updated. A batch size of 32 random *K* bit message blocks *M* is generated prior to performing backpropagation on the end-to-end version of the model, with the channel model acting as the true channel proxy. The procedure is repeated until convergence, which is indicated by the validation loss (a validation batch size of 32 random *K* bit message blocks is used to measure this loss for the stopping condition). This process is repeated for 1600 steps in each training epoch with up to a maximum of 300 epochs. After a single epoch, the simulated channel block error rate (BLER_sim_) is calculated against the proxy channel model for monitoring purposes. In our experiments, we have also calculated BLER against the true channel to measure the correlation between the BLER_sim_ and the BLER. We observe that while the BLER_sim_ has higher variance, it is well correlated with the BLER and is a suitable indication of expected model performance at the current SNR of the channel. In our experiments, we trained on a simulated channel at an SNR of 6 dB.

### 3.5. Simulated Channel Environments

To investigate the performance of the model, we train on four instantaneous channel functions, an AWGN channel, a Rayleigh fading channel, a Rician channel with Rician factor equal to 4, and a non-linear power amplifier with an additive white Gaussian Noise (PA-AWGN) channel. Each of these functions includes an additive noise component as shown in the AWGN channel Equation (5).

(5) r(t)=z(t)+n(t)

The Rayleigh and Rician fading channels scale the transmitter symbols z(t) with fading coefficients a(t) Equation (6). However, they each differ in how the fading coefficients are calculated.

(6) r(t)=a(t)z(t)+n(t)

In the Rayleigh fading channel, the fading coefficients are drawn from a complex standard normal distribution a∼CN(0,1) and their argument is scaled and multiplied with a phased waveform $a\left(t\right)=\frac{1}{\sqrt{2}}\left|a\right|e^{j\psi }$, in our case we assume a zero phase ψ=0.

The Rician fading channel function r(t) in Equation (6) draws its scaling coefficients from a complex normal distribution with parameters μ and σ, $a∼CN(\mu ,\sigma^{2})$. The mean $\mu =\sqrt{\frac{K}{\left(2(K+1)\right)}}$ and standard deviation $\sigma =\sqrt{\frac{1}{\left(2(K+1)\right)}}$ are parameterised by the Rician factor *K*, which we define as K=10. The lower the value for *K*, the Rician fading appears to become similar to Rayleigh fading, and for higher values of *K*, Rician fading resembles AWGN.

In the PA-AWGN channel, we apply a solid-state high-power amplifier (SSPA) to translate transmitter symbols prior to the AWGN channel. Equation (7) shows the Rapp model [29] with parameters for the limiting output amplitude $A_{0}$, amplifier gain ν, and smoothness *p*. In our experiments, these are set to $A_{0}=1$, ν=1, and p=5. The transmitter symbols are then transformed by the amplifier function, as shown in Equation (8).

(7) $$g\left(A\right)=\nu \frac{A}{{\left(1+{\left[{\left(\frac{\nu A}{A_{0}}\right)}^{2}\right]}^{p}\right)}^{1/2p}}$$

(8) $$z^{\prime }\left(t\right)=g\left(|z\left(t\right)|\right)e^{j∠z(t)}$$

Since in this article we simulate the channel, we parameterise the function with the ratio of energy per information bit to the noise power spectral density $E_{b}/N_{0}$ in dB and a parameter for the code rate K/N. We use the code rate to convert to the ratio for the energy per symbol to noise power spectral density $E_{s}/N_{0}\;\mathrm{dB}=E_{b}/N_{0}\;\mathrm{dB}+10log_{10}\left(K/N\right)$ and use the linear form $E_{s}/N_{0}=10^{E_{s}/N_{0}\;\mathrm{dB}/10}$ to estimate the separate components $E_{s}=\sum_{t=1}^{L}z{\left(t\right)}^{2}/L$ and $N_{0}=E_{s}/\left(E_{s}/N_{0}\right)$. The noise variance $\sigma^{2}=N_{0}/2$ is then used to draw the complex Gaussian noise parameter $n\left(t\right)∼CN\left(0,\sigma^{2}\right)$. Once the noise is determined, it can be applied to the channel transfer function.

We trained and evaluated our proposed method and the joint end-to-end method against each of these channels. The joint approach is based on the model defined in [1] and includes the instantaneous channel transfer function as part of the network architecture. In this approach, there is no requirement for an iterative training algorithm, and the training is performed by backpropagation over a maximum of 300×12 epochs, with a batch size of 32 K=8 bit messages. The Adam optimisation algorithm [30] is applied in both approaches, and we leverage stochastic weight averaging (SWA) [31] every 10 epochs with a cyclical learning rate schedule [32] having a minimum learning rate of 0.0001 and maximum of 0.001.

## 4. Results and Discussion

While the baseline end-to-end joint and proposed iterative methods take different approaches to training, once trained, the transmitter and receiver can be separated from the end-to-end model and deployed separately for testing. In the iterative method, the channel model is not required for deployment and is used only during training. Both approaches are evaluated by transmitting generated random K=8 bit message blocks and transmitting over each of the simulated channel transfer functions. The BLER is calculated for each block at varying SNR between 0 to a maximum of 15 dB. In this section, we present results for both methods, as well as the uncoded BLER maximum likelihood decoding performance.

**Figure 6:**
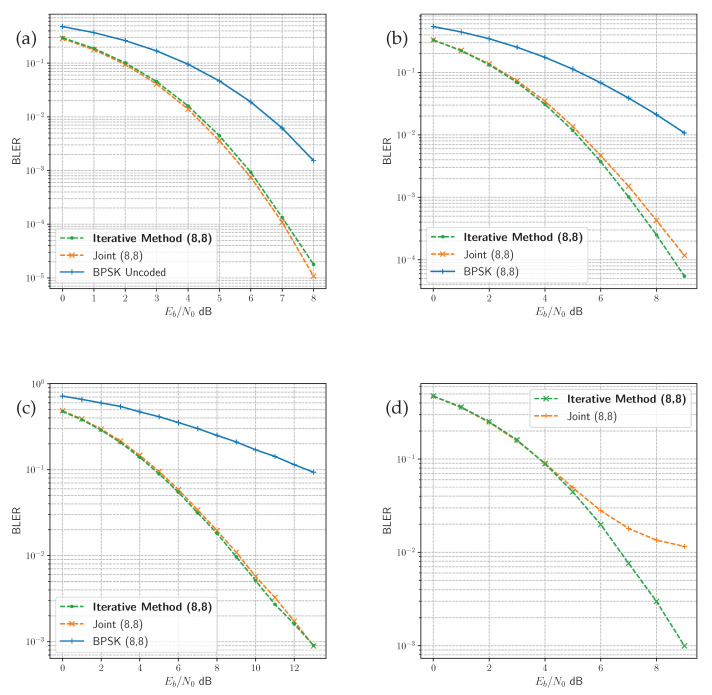
Comparison of BLER performance in the four channel environments. Uncoded K=8 bit BPSK modulation is compared with the joint and the proposed iterative method in the (**a**) AWGN channel, (**b**) Rician fading channel, (**c**) Rayleigh fading channel, and joint and iterative methods are compared in (**d**) the PA-AWGN channel.

The performance of both methods under each channel is presented in Figure 6, and is compared with uncoded BPSK for reference. Even though the proposed iterative method has been trained on a generated model, while the joint method is trained with an assumed channel function, there is very little difference between the performance of both. The PA-AWGN channel is an exception, however. The proposed method outperforms the joint method, which appears to have an error floor in higher SNR. Each of the DL methods achieves gains over the uncoded BPSK modulation. This is because the uncoded BPSK modulation represents the 8 bit sequence with 8 symbols, each chosen from one of two constellation points. For example, −1+0j for 0 and 1+0j for 1. Whereas the DL methods can map each one of the $2^{K}$ messages to any arrangement of 8 symbols in the IQ space. The DL methods learn this mapping by minimising the error in message recovery subject to the distortions introduced by the channel.

The joint end-to-end model has been trained with full information of the simulated channel environment, due to the assumed channel layer. In contrast, our proposed approach trains a separate channel model to act as a proxy for the true channel environment, given observations of random noise. The RL, GAN and diffusion approaches outlined in the literature compare solutions with variants of the canonical joint end-to-end learning method [4,7,9,11,13,14,15]. This is to demonstrate equivalent or better performance against the model, which is trained with the assumed channel function. Doing so indicates that the method learns an optimised code based on the observations without prior knowledge of the channel. The BLER performance for our proposed approach indicates that the resulting channel model provides an accurate representation of the true channel environment. This approximation enables the transmitter model to learn an optimised code for the target channel environment.

**Figure 7:**
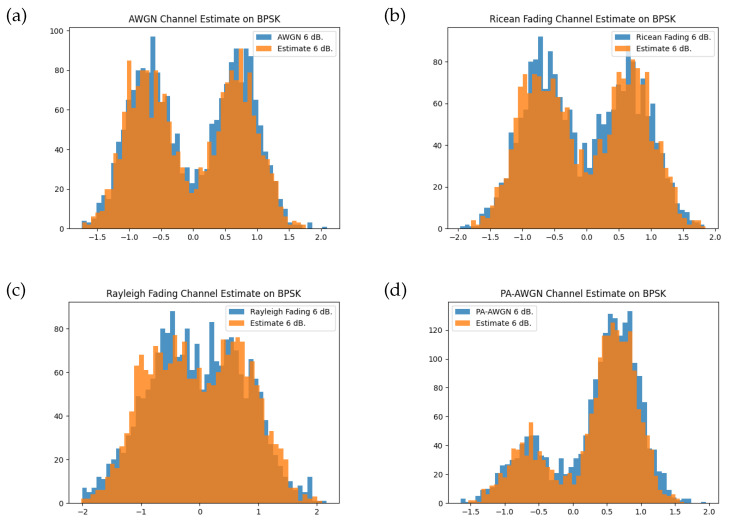
Histogram of channel symbols for each instantaneous channel function and the trained channel model at training SNR of 6 dB for random BPSK modulation. Comparisons are shown for the (**a**) AWGN channel, (**b**) Rician fading channel, (**c**) Rayleigh fading channel, and (**d**) PA-AWGN channel.

During the training of the channel model, the origin transmitter samples are drawn from the random uniform distribution s(t)∼U([−1,1]) prior to transmitting over the instantaneous channel function. The channel model does not learn from an information-carrying modulation, as such it does not learn unique features specific to a given waveform. While this could be a disadvantage, the BLER performance indicates that the channel model provides a suitable approximation that enables the transmitter and receiver to jointly learn an appropriate representation for the transmit symbols. In our evaluation, we review the channel effect on a BPSK modulation and compare this with the estimates produced by the channel model. The channel model is able to approximate distributions of the instantaneous channel as shown in Figure 7. The intention of training on uniform IQ samples is to prevent transmission of an intelligible information-carrying signal during the training procedure. The resulting bimodal distribution for each channel function with the BPSK modulation is approximated well by the trained channel model, which produces a mixture of Gaussians with different scales and locations corresponding to the two modulation symbols.

**Figure 8:**
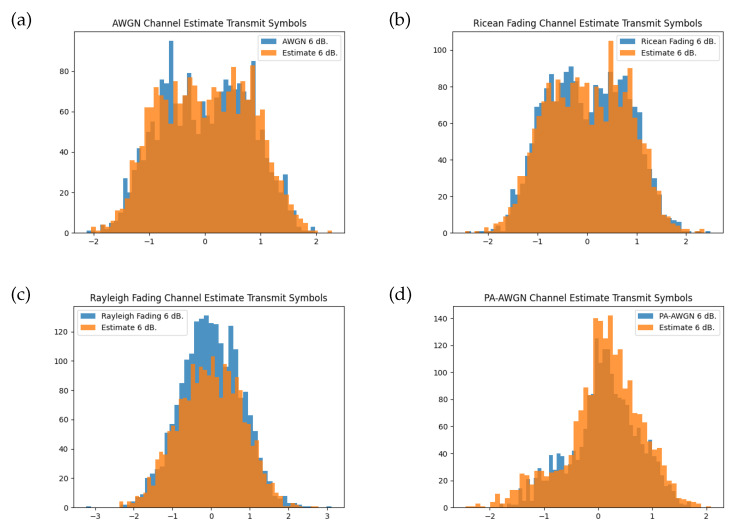
Histogram of channel symbols for each instantaneous channel function and the trained channel model at training SNR of 6 dB for K=8 bit messages transferred through the transmitter model. Comparisons are shown for the (**a**) AWGN channel, (**b**) Rician fading channel, (**c**) Rayleigh fading channel, and (**d**) PA-AWGN channel.

The transmitter model, however, does not learn a conventional modulation; instead, the transmit symbols make use of the IQ space more broadly. Figure 8 shows the histogram for the instantaneous channel transfer function and the approximation given by the channel model when provided with the learnt transmitter symbols. The channel model approximation is close to that of the true distribution when presented with a non-uniform modulation.

**Figure 9:**
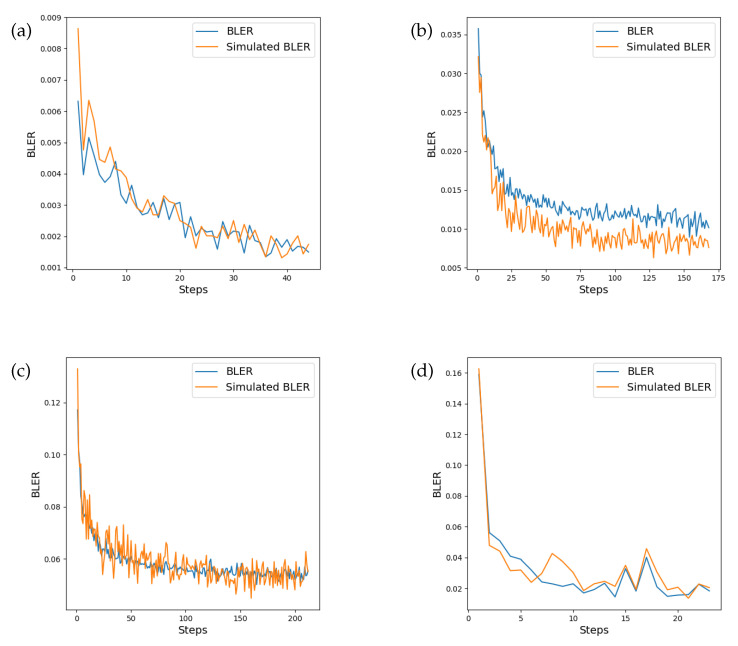
Comparison between BLER performance on the instantaneous channel transfer function and the simulated channel model recorded after each epoch of training. There is a high correlation between BLER between the true transfer function and the simulated channel model. The monitored BLER is shown in the (**a**) AWGN channel, (**b**) Rician fading channel, (**c**) Rayleigh fading channel, and (**d**) PA-AWGN channel.

The question of when to stop training often relies on monitoring a performance metric such as the validation loss, and once the metric ceases to decrease after a fixed number of steps, the training cycle ceases. However, when the intention is to carry out training without feedback over the true channel, training metrics may no longer be reliable for determining whether the end-to-end system is learning under the true channel conditions. Outside of the negative log-likelihood loss for the channel, it is desirable to be able to monitor a performance metric which is a good indicator of the training progress of the end-to-end system. Our intuition is that if the channel model is learning an accurate representation of the true channel transfer function, the BLER produced by evaluation of transmitter and receiver via the channel model should reflect the BLER that would be produced over the true transfer function. Evaluation of the transmitter and receiver was performed on both the instantaneous channel transfer function as well as the channel model at the end of each epoch. Figure 9 shows the monitored value of the BLER during training at the fixed SNR of 6 dB. We note that, in general, the BLER corresponds well with that recorded on the true channel, apart from the Rician fading channel, where the simulated BLER is lower. However, the error signal correlates well in each channel and serves as a suitable proxy measure during training (Table 4). It is also worth observing that the variance of the BLER differs between the true and simulated channels. This is more visible in the Rician and Rayleigh fading channels, which have a larger number of training epochs.

In the field, evaluation of the true channel function may not be feasible after each epoch, hence monitoring performance will be reliant on the accuracy of the simulated channel model. If monitoring of the true channel performance is required, it is possible to intermittently deploy the transmitter weights to the origin side to evaluate performance at irregular intervals rather than every epoch. This is to decrease the frequency at which information-carrying transmissions are made during the training cycle and to maintain burst communications decreasing the chance of intercept.

Generative models provide a suitable method for enabling backpropagation in OAL, but the GAN method has been the subject of much research for learning in wireless communication systems without an assumed channel. Instead of concentrating on the GAN approach, we have instead proposed a simpler generative model capable of modelling the channel output distributions as shown in our results. By demonstrating equivalent performance to the joint end-to-end model, we are comparing our results to a model that has full knowledge of the simulated channel environment. In doing so, we demonstrate that the use of the MDN can provide a sufficient approximation of the true channel environment to permit the learning of an optimal code for that environment.

## 5. Limitations

As a generative model. the MDN is still vulnerable to mode collapse. Reasons for mode collapse in the MDN model are described in [33], suggesting that the primary reason is due to an imbalanced representation of data associated with modes in the training set. The authors suggest that the mixture components associated with the dominant modes represented in the data outweigh the other mixture coefficients and prevent variation in learning solutions [33]. They introduce additional loss terms that help to penalise large value weights and high variance parameters [33]. Our results did not suffer from mode collapse. One limitation of our approach is that we are using a small code size, and we are simulating a memoryless channel environment. In a real channel environment, certain channel states may persist for longer and therefore become over-represented during the training phase for the channel model. In a physical system, it may be more likely to encounter mode collapse and would require an exploration of approaches to mitigate this issue.

Additional limitations of our work include the assumption of ideal synchronisation. While the work focuses on the use of the MDN as a simpler alternative to generative modelling, scope remains for testing in more complex channels, which include timing, phase, and frequency offsets. These would lead to the additional considerations of a AE architecture suited to learning matched filtering and compensating for the additional channel effects. In keeping the experiments simple, we have also restricted our work to a short message length of K=8 bits rather than investigating extension to longer codes.

The removal of the feedback channel has reduced the opportunity for data poisoning between the transmitter and receiver during training. However, there is still some potential for data poisoning during stage 2 of the learning procedure, when random uniform IQ symbols are transmitted over the true channel environment. This phase occurs as a regular burst transmission during each training iteration. While it may be possible to mitigate somewhat by reducing the regularity of this phase, the training of the channel model is reliant on sampling of uniform noise through the true channel environment.

## 6. Conclusions and Future Work

### 6.1. Conclusions

In this article, we have proposed an alternate generative channel model for training of transmitter and receiver OAL without relying on a feedback channel. We have shown that the MDN is able to model the distribution of a stochastic channel environment. As indicated by our results (Section 4), the proposed approach is able to produce an equivalent BLER to the joint end-to-end model, without a prior assumption of the channel function. We have demonstrated equivalent BLER performance for the K=8 bit uncoded message in the AWGN, Rician fading, and Rayleigh fading channels as well as in the PA-AWGN channel. This is achieved without the need for a fixed channel model. Prior work has focused on the GAN model to learn the channel distribution, and while this has been shown to be effective, the training procedure does add complexity. We have demonstrated that the simpler MDN model, requiring only one Gaussian distribution per channel symbol, is a capable replacement for the GAN when modelling memoryless channels and does not require the overhead of a discriminator model during training. We have also shown that intermittent sampling of the end-to-end performance in terms of BLER is suitable for use as a stopping condition during the training process. This allows training to occur entirely on the receiver side without the need for feedback over the true channel, such as in RL based methods. The MDN is advantageous for OAL learning, does not require complex training regimes, and does not require multiple forward passes during inference (such as in the diffusion model). Removal of the feedback channel prevents the opportunity for data poisoning via feedback during the training process. The proposed approach is able to approximate the channel distribution such that the transmitter and receiver were able to learn an optimal code matching the performance of the joint end-to-end model.

### 6.2. Future Work

The limitations identified in Section 5 present opportunities for future work. Application to physical channel environments would necessarily require the addition of synchronisation. This would require modifications to both the transmitter and receiver architectures to support filtering and the ability to correct timing, phase, and frequency offsets in the receiver. To this end, it is possible to investigate extending the AE-based architecture to learn filtering, detection, and synchronisation. Conventional methods for synchronisation have been optimised for specific forms of filtering and modulation, yet it may be possible to combine data-driven and conventional approaches, such as in the work on deep unfolding [34]. In addition, physical channels may exhibit a certain degree of memory, as identified in [33], mode collapse in MDN is due to unequal representation of states during training. Future work will need to investigate the properties of channels with memory and investigate the effects on the MDN channel model as well as investigate methods to mitigate the unbalanced representation of states within the training data. While short codes have application in resource-constrained devices such as in the IoT, future work would also be required to make suitable modifications to the transmitter and receiver architectures to support longer codes or integrate with concatenated coding methods. Scalability due to message length and the challenges posed both to the architecture and to the sampling requirements of the learning process are areas that require further work for the practical application of DL methods to trainable wireless communications systems. Although the feedback path is no longer a vulnerability, there remains some potential for data poisoning the forward path during burst transmissions in stage 2 of training. Future work should investigate methods to mitigate this potential vulnerability by reducing the frequency of transmission, employing a low probability of detecting signalling as well as experimentally investigating robustness of the proposed method to data poisoning attacks. SDR platforms offer flexibility for defining novel wireless communications systems. The emergence of embedded and edge device hardware platforms supporting the optimisation required for the parallel computations required by DL will be necessary for translation of DL-based methods from software experimentation to hardware implementation. However, trainable wireless communication systems introduce a new paradigm where components of the system are no longer static and must support methods for redeployment and reconfiguration during operation.

## Figures and Tables

**Table 1:** The transmitter consists of four groups, input, skip block, a linear transformation, and an output block. The number of units is specified for the dense layers, batch normalisation, and swish activation preserve the same dimension of output as produced by the dense layer. The model was trained to map an uncoded message of K=8 bits to N=8 IQ symbols.

Layer	Units Uncoded 8 Bit	Group
Input layer	$2^{K}$	Input
Dense layer	512	Skip block
Batch normalisation	-	
Swish activation	-	
Dense layer	64	
Batch normalisation	-	
Swish activation	-	
Dense layer	512	
Batch normalisation	-	
Swish activation	-	
Dense layer	2N	2N linear block
Linear activation	-	
Reshape [N,2] layer	-	
Dense layer	2	Output [N,2]
Tanh activation	-	
Energy normalisation	-	

**Table 2:** The receiver network consisted of three groups for input, feature learning (skip block), and output. The dimensions of units are shown for each dense layer with subsequent layers producing the same shape output as the preceding dense layer. The receiver was trained to map N=8 IQ symbols to the original K=8 bit message.

Layer	Units Uncoded 8 Bit	Group
Input layer	[N,2]	Input
Flatten layer	-	
Dense layer	512	Skip block
Batch normalisation	-	
Swish activation	-	
Dense layer	64	
Batch normalisation	-	
Swish activation	-	
Dense layer	512	
Batch normalisation	-	
Swish activation	-	
Dense layer	$2^{K}$	Output
Softmax activation	-	

**Table 3:** The channel model receives as input the transmitter symbols z(t) and learns to approximate the distribution for the true instantaneous channel function r(t). The final layers learn the parameters for the mean and standard deviation of a normal distribution around each IQ symbol in r(t).

Layer	Units Uncoded 8 Bit	Group
Input layer	[N,2]	Input
Dense layer	512	Skip block
Batch normalisation	-	
Swish activation	-	
Dense layer	64	
Batch normalisation	-	
Swish activation	-	
Dense layer	512	
Batch normalisation	-	
Swish activation	-	
Dense layer	512	Skip block
Batch normalisation	-	
Swish activation	-	
Dense layer	64	
Batch normalisation	-	
Swish activation	-	
Dense layer	512	
Batch normalisation	-	
Swish activation	-	
Dense layer	[N,4]	Distribution Parameters
Mean branch	[N,2]	
Standard Deviation branch	[N,2]	
Mean Linear activation	-	
Standard Deviation Softplus activation	-	
Sample Normal distribution	[N,2]	Output

**Table 4:** Pearson $\rho_{c}$ and Spearman’s rank $\rho_{s}$ correlation coefficients for the BLER produced on true and simulated channels. The high correlation as well as the error curves indicates a suitability for the simulated channel model to act as a proxy for performance monitoring during training as well as acting as a metric for the training stopping condition.

Channel Type	$\rho_{c}$	$\rho_{s}$
AWGN	0.94	0.89
Rician Fading	0.94	0.73
Rayleigh Fading	0.88	0.66
PA-AWGN	0.99	0.93

## Data Availability

Data is contained within the article.

## Abbreviations

The following abbreviations are used in this manuscript:

Adam	adaptive moment estimation
AE	autoencoder
AWGN	additive white Gaussian noise
BLER	block error rate
BLER_sim_	simulated channel block error rate
BPSK	binary phase shift keying
CE	cross-entropy
CNN	convolutional neural network
DDIM	denoising diffusion implicit model
DDPM	diffusion-denoising probabilistic model
DL	deep learning
GAN	generative adversarial network
IoT	internet of things
IQ	in-phase and quadrature
MDN	mixture density network
NLL	negative log-likelihood
OAL	over-the-air learning
PA-AWGN	power amplifier with an additive white Gaussian noise
QPSK	quadrature phase-shift keying
RA-GAN	residual aided generative adversarial network
ReLU	rectified linear unit
RL	reinforcement learning
SDR	software-defined radio
SISO	single input single output
SNR	signal to noise ratio
SPSA	simultaneous perturbation stochastic approximation
SSPA	solid-state high-power amplifier
SWA	stochastic weight averaging
WGAN	Wasserstein generative adversarial network

## References

[1] O’Shea T., Hoydis J. (2017). An Introduction to Deep Learning for the Physical Layer. IEEE Trans. Cogn. Commun. Netw..

[2] Raj V., Kalyani S. (2018). Backpropagating through the Air: Deep Learning at Physical Layer without Channel Models. IEEE Commun. Lett..

[3] Aoudia F.A., Hoydis J. End-to-End Learning of Communications Systems without a Channel Model. Proceedings of the 2018 52nd Asilomar Conference on Signals, Systems, and Computers.

[4] Ye H., Li G.Y., Juang B.F., Sivanesan K. Channel Agnostic End-to-End Learning Based Communication Systems with Conditional GAN. Proceedings of the 2018 IEEE Globecom Workshops (GC Wkshps).

[5] O’Shea T.J., Roy T., West N. Approximating the Void: Learning Stochastic Channel Models from Observation with Variational Generative Adversarial Networks. Proceedings of the 2019 International Conference on Computing, Networking and Communications (ICNC).

[6] Sagduyu Y.E., Shi Y., Erpek T. (2021). Adversarial Deep Learning for Over-the-Air Spectrum Poisoning Attacks. IEEE Trans. Mob. Comput..

[7] Dörner S., Henninger M., Cammerer S., Brink S.T. WGAN-based Autoencoder Training Over-the-air. Proceedings of the 2020 IEEE 21st International Workshop on Signal Processing Advances in Wireless Communications (SPAWC).

[8] Davey C.P., Shakeel I., Deo R.C., Salcedo-Sanz S. (2023). Channel-Agnostic Training of Transmitter and Receiver for Wireless Communications. Sensors.

[9] Dörner S., Cammerer S., Hoydis J., Brink S.T. (2018). Deep Learning Based Communication Over the Air. IEEE J. Sel. Top. Signal Process..

[10] Cammerer S., Aoudia F.A., Dörner S., Stark M., Hoydis J., Brink S.T. (2020). Trainable Communication Systems: Concepts and Prototype. IEEE Trans. Commun..

[11] Ye H., Liang L., Li G.Y., Juang B.H. (2020). Deep Learning-Based End-to-End Wireless Communication Systems with Conditional GANs as Unknown Channels. IEEE Trans. Wirel. Commun..

[12] Alawad M.A., Hamdan M.Q., Hamdi K.A. (2022). Innovative Variational AutoEncoder for an End-to-end Communication System. IEEE Access.

[13] Jiang H., Bi S., Dai L., Wang H., Zhang J. (2022). Residual-Aided End-to-End Learning of Communication System without Known Channel. IEEE Trans. Cogn. Commun. Netw..

[14] Kim M., Fritschek R., Schaefer R.F. (2023). Learning End-to-End Channel Coding with Diffusion Models. Proceedings of the WSA & SCC 2023; 26th International ITG Workshop on Smart Antennas and 13th Conference on Systems, Communications, and Coding.

[15] Aoudia F.A., Hoydis J. (2019). Model-free training of end-to-end communication systems. IEEE J. Sel. Areas Commun..

[16] Goodfellow I. (2016). Nips 2016 tutorial: Generative adversarial networks. arXiv.

[17] Arjovsky M., Chintala S., Bottou L. Wasserstein generative adversarial networks. Proceedings of the International Conference on Machine Learning, PMLR.

[18] Bishop C.M. (1994). Mixture Density Networks. Technical Report, Aston University. https://research.aston.ac.uk/en/publications/mixture-density-networks.

[19] Unni R., Yao K., Zheng Y. (2020). Deep Convolutional Mixture Density Network for Inverse Design of Layered Photonic Structures. ACS Photonics.

[20] Karoliny J., Etzlinger B., Springer A. Mixture Density Networks for WSN Localization. Proceedings of the 2020 IEEE International Conference on Communications Workshops (ICC Workshops).

[21] Mostafavi S., Sharma G.P., Gross J. Data-Driven Latency Probability Prediction for Wireless Networks: Focusing on Tail Probabilities. Proceedings of the GLOBECOM 2023–2023 IEEE Global Communications Conference.

[22] Khurjekar I.D., Gerstoft P. Multi-Source DOA Estimation With Statistical Coverage Guarantees. Proceedings of the ICASSP 2024–2024 IEEE International Conference on Acoustics, Speech and Signal Processing (ICASSP).

[23] Li H., Kanuric T., Eichberger A. (2023). Automotive Radar Modeling for Virtual Simulation Based on Mixture Density Network. IEEE Sensors J..

[24] Ioffe S., Szegedy C. Batch Normalization: Accelerating Deep Network Training by Reducing Internal Covariate Shift. Proceedings of the 32nd International Conference on Machine Learning.

[25] Ramachandran P., Zoph B., Le Q.V. (2017). Searching for activation functions. arXiv.

[26] Veit A., Wilber M.J., Belongie S. (2016). Residual networks behave like ensembles of relatively shallow networks. Adv. Neural Inf. Process. Syst..

[27] Dubey S.R., Singh S.K., Chaudhuri B.B. (2022). A Comprehensive Survey and Performance Analysis of Activation Functions in Deep Learning. Neurocomputing.

[28] Glorot X., Bordes A., Bengio Y. Deep sparse rectifier neural networks. Proceedings of the Fourteenth International Conference on Artificial Intelligence and Statistics, JMLR Workshop and Conference Proceedings.

[29] Rapp C. (1991). Effects of HPA-nonlinearity on a 4-DPSK/OFDM-signal for a digital sound broadcasting signal. ESA Spec. Publ..

[30] Kingma D.P., Ba J. (2014). Adam: A method for stochastic optimization. arXiv.

[31] Izmailov P., Podoprikhin D., Garipov T., Vetrov D., Wilson A.G. (2018). Averaging weights leads to wider optima and better generalization. arXiv.

[32] Smith L.N. Cyclical Learning Rates for Training Neural Networks. Proceedings of the 2017 IEEE Winter Conference on Applications of Computer Vision (WACV).

[33] Zhou Y., Gao J., Asfour T. (2020). Movement primitive learning and generalization: Using mixture density networks. IEEE Robot. Autom. Mag..

[34] Jagannath A., Jagannath J., Melodia T. (2021). Redefining Wireless Communication for 6G: Signal Processing Meets Deep Learning With Deep Unfolding. IEEE Trans. Artif. Intell..

